# Parasitology and One Health—Perspectives on Africa and Beyond

**DOI:** 10.3390/pathogens10111437

**Published:** 2021-11-05

**Authors:** Vincenzo Lorusso

**Affiliations:** 1 Global Research & Intellectual Property, Vetoquinol, 37 Rue de la Victoire, 75009 Paris, France; V.Lorusso@salford.ac.uk or vincenzo.lorusso@vetoquinol.com; 2 University of Salford Tick Infections (USALTI)-Afrique, School of Science, Engineering & Environment, University of Salford, Greater Manchester, Salford M5 4WT, UK; 3 African Institute of One Health Research and Diagnostics (AIOHRD), University of Abuja, km 23 Airport Road, Abuja 900110, Nigeria

**Keywords:** Africa, One Health, zoonoses, parasitology, vector-borne diseases, vectors, international cooperation, research & innovation, education

## Abstract

This concept paper reviews issues pertaining to parasitic and vector-borne infections, of humans, animals, or both, of topical relevance to the African continent as well as to neighbouring and interconnected geographies. This analysis is carried out through the “One Health” lens, being mindful of the central role of agriculture and livestock keeping in Africa’s sustainable development. The possible agricultural transformation that the continent may undergo to fulfil the rising demand for animal protein of its growing population, coupled with the ongoing climate changes, may lead to potentially enhanced interactions among humans, domesticated and wild animals, in a fast-changing environment. In this view, tackling parasitic conditions of livestock can prove being multidimensionally beneficial by improving animal health as well as communities’ food security, livelihood and public health. Accordingly, the value of applying the One Health approach to drug discovery and development in the fight against parasitic neglected tropical diseases and zoonoses, is also underscored. Overall, this article upholds the adoption of a holistic, global, interdisciplinary, multisectoral, harmonised and forward-looking outlook, encompassing both life and social sciences, when dealing with parasitic conditions of humans and animals, in Africa and beyond, in COVID-19 times and further.

## 1. Introduction

### 1.1. Africa, Present and Future

With nearly 60% of its population (~800 million) under the age of 25 years, Africa is the “youngest” continent on earth [1]. Currently hosting a total of 1.37 billion people [2], corresponding to almost a double of Europe’s 750 million, the continent is expected to reach the size of 2.5 billion inhabitants by 2050 and of approximately 4 billion by 2100 [3]. By then, one person in every three worldwide will be from the African continent. This exponential growth is expected to be accompanied by an equally significant increase in the continent’s need in animal source foods. Indeed, Africa’s demand for meat, milk and eggs will almost quadruple by 2050 [4,5], with annual growth rates of consumption estimated at 2.3% for milk and 2.8% for meat (with beef and poultry being Africa’s most consumed meats) [6]. Currently, however, not only Africa is overall a net food importer [7,8], but it is also the most food insecure region in the world, the only one in which the absolute number of undernourished people has increased in the past 30 years (282 million in 2020) [9,10], and where that of stunted children under five is still rising (61.4 million in 2020) [10,11]. Presently, nearly 60% of Africans (798.8 million) are moderately or severely food insecure, with more than 90% (724.4 million) of them residing in sub-Saharan Africa [10]. At the same time, overweight rates are also increasing [12], highlighting the continent’s need for nutritious foods.

Yet, Africa harbours 60% of the world’s uncultivated arable land (~600 million hectares) [8,13,14], with one-quarter of the world’s cultivable land being in sub-Saharan Africa but only producing 10% of the global agricultural output [15]. Therefore, the continent’s rising demand for animal protein could be potentially met, at least partly, through enhanced local agricultural production, made possible by improving the productivity of farming processes (e.g., through land and water use optimisation and through the development of local transformative value chains) [8,13], as envisaged by the African Union’s Comprehensive Africa Agriculture Development Programme (CAADP) [16] and New Alliance for food security and nutrition [17]. This agricultural transformation process may also include possible access by livestock keepers to wild areas hardly attended or inhabited thus far, to make room for livestock rearing and grazing [18]. By creating new human–animal–environment interfaces, such an expansion may however bring health risks, as pathogens from wildlife could spill over onto domestic animals and people [18]. Moreover, the ongoing climatic changes and global warming may also compound this scenario. Indeed, the spread of desertification threatening several African regions such as the Sahel and the Horn of Africa [19], may cause the potential concentration of livestock keeping in certain areas, in the form of more intensified (than presently) livestock rearing, conceivably increasing land erosion [13,20]. Furthermore, Africa’s ongoing vertiginous urbanisation at a 3.5% yearly rate [21] is also expected to contribute to the convergence of livestock and people on urban and peri-urban areas in the coming decades [22]. This would provide intensified occasions of contact between humans, domesticated and wild animals, thereby creating augmented opportunities for the emergence and transmission of infectious diseases and zoonoses^1^ [22,23,24]. Altogether, this will require an enhanced surveillance and monitoring of livestock and environmental health, including wildlife movement and fitness, biodiversity richness, as well as use and management of water, land cover and vegetation.

### 1.2. COVID-19, Agriculture and Livestock Keeping

Although with a certain degree of variability according to countries, overall Africa has so far been hit by a lower number of COVID-19 cases and fatalities compared to other continents of the world [25,26,27]. However, the pandemic has still shown to be a major source of hindrance, especially during the first wave of lockdowns in 2020, when the setbacks of international trade caused serious disruptions in food value chains and supplies [28,29]. Under COVID-19, Africa’s food security has also been further weakened due to income reductions and food price inflation, outcomes of lower availability of agricultural labour and produces, reduced liquidity for traders and interruptions of social protection programmes [30,31]. In 2020, the number of Africans facing hunger increased by 3%, with approximately 46 million more undernourished people being recorded compared to the previous year [10]. In Nigeria, the biggest African economy by nominal GDP [32], for instance, 75.5% of the adult population became moderately or severely food insecure (from 48.5% in 2018–2019) and 33.5% severely food insecure (from 14% in 2018–2019). Urban households were significantly more food insecure, suggesting their higher vulnerability compared to rural areas, following the onset of the pandemic [33]. This further highlights the fragility of the African net food importing model.

Against this background, the role of agricultural development will be absolutely crucial for Africa to rebound. Agriculture, including also livestock keeping, is indeed still the largest economic sector in the continent, accounting on average for 15% of Africa’s GDP, ranging from below 3% in Botswana and South Africa to over 50% in Chad [31,34]. Agriculture employs 60% of Africa’s working age population [8], with at least 50% of the workforce being represented by women [35]. Indeed, despite the intense ongoing urbanisation, a significant proportion of Africa’s population (e.g., 60% in sub-Saharan Africa and 40% in Northern Africa) still resides in rural areas [36], and oftentimes migrations to the cities are followed by returns to the country, according to a “circular” pattern [37]. Within this context, family-owned smallholder farms account for approximately 80% (i.e., 33 million) of all farms in Africa [38] and contribute up to 70% of the continent’s food supply [14]. The term of “smallholder” farms, usually interchanged with “small-scale agriculture”, “family farms”, “subsistence farms”, “resource-poor farm”, “low-income farms”, “low-input farm” or “low-technology farm” [39,40], refers to contexts “operating under structural constraints such as access to sub-optimal amounts of resources (e.g., capital and assets), technology and markets” [40,41], and “in less than 2 hectares of cropland” [42]. Although on the rise in some countries, medium-scale commercial farms (5–100 hectares), or “agribusiness”, still represent an overall minority in the continent’s agricultural landscape [43,44].

Livestock keeping is an essential component of Africa’s agriculture, with 66% of Africa’s land being used to graze animals [45]. The majority (i.e., over two-thirds) of Africa’s smallholder farmers engage in livestock keeping (i.e., 44% in Nigeria up to 79% in Niger), not only sustenance for, but also for livelihood, manure and traction [6]. In addition to sedentary mixed crop-livestock farming, pastoralism is a key feature of the continent’s livestock keeping, including 38 million herders [46,47] and representing the main subsistence for an estimated 268 million people [48]. Practised in 43% of Africa’s land mass, it is the dominant livelihood system in drylands, from the Sahel to the Horn of Africa, spanning also parts of Southern Africa [48]. Contributing 10–44% of GDP of African economies, pastoralism displays the unique ability to add value and convert scarce natural resources into animal source food and income, providing approximately 90% of the meat consumed in East Africa and nearly 60% of the meat and milk products consumed in West Africa [49]. The mobile transhumant or nomadic lifestyle, which is the essence of pastoralism, often entailing cross-border movements, frequently exposes pastoralists to social conflicts and violence, insecure land rights and access, vulnerability to climate change and deteriorating natural resources as well as infectious and zoonotic diseases [46,47,50,51]. As a result, 13.4 million pastoralists in sub-Saharan Africa live in conditions of extreme poverty [47,50]. Poor livestock keepers are indeed particularly exposed to zoonoses due to their recurrent contacts with livestock, their consumption of often poorly processed animal produces and their limited access to health provision, both for themselves and their animals [50]. Furthermore, the majority of animal food sources and livestock in Africa are traded in traditional and wet markets, which play a key role in Africa’s societies and agricultural landscapes, not only as a source of nourishment and income, but also for social and cultural cohesion [6,52]. With their number expected to grow to meet the increasing demand for foodstuffs, accrued efforts will be needed to ensure food and livestock markets in Africa will fulfil safety and quality standards in the interest of consumers and global public health [52].

All in all, the ongoing steep demographic growth, coupled with the COVID-19 crisis showing the unsustainability of Africa’s net food importing model, will likely require, as a response, the enhancement of agricultural and livestock keeping activities in the continent [30]. Indeed, investments in smallholder agriculture in Africa can lead to multidimensional benefits such as increasing food production and food security, poverty reduction [14] and employment opportunities for Africa’s burgeoning working age population, allowing the continent to harness its demographic dividend^2^ [53]. In sub-Saharan Africa only, economic growth from agriculture is indeed estimated to be 11 times more effective at reducing extreme poverty than any other sector [14].

Already intimate in the essence of smallholder farming, the human–animal bond and interaction will still be at the centre and may be further enhanced in the years to come, as it will play a critical role in securing Africa’s sustainable development. This will render the need for surveilling and monitoring zoonotic infections ever topical in the continent, and across all neighbouring, trading and partner geographies.

## 2. One Health and Parasitic and Arthropod-Borne Infections

In a world dominated by the COVID-19 pandemic, the wording of “One Health” is now widely known and employed, even beyond the scientific community. One Health is defined, according to the Tripartite collaboration between the Food and Agriculture Organization of the United Nations (FAO), the World Organisation for Animal Health (OIE) and the World Health Organization (WHO), as “a collaborative, multidisciplinary, and multisectoral approach that can address urgent, ongoing, or potential health threats at the human–animal–environment interface at subnational, national, global and regional levels” [54]. This stems from the realisation that (i) the health of human beings and animals are interdependent on each other and on the health of the ecosystems they inhabit and that (ii) approximately 60% of human infections and 75% of newly emerging infections originate from an animal source [55]. The global recognition of the importance of this approach is also reflected in the G20 Rome Declaration from the recent Global Health Summit, held in May 2021 [56].

The locution of One Health is undoubtedly not novel to parasitologists^3^, who, by vocation, study infections and their causative agents and hosts, at the interface with human and veterinary medicine. With special regards to parasitic conditions, Africa harbours a plethora of them that require to be addressed according to the One Health approach, due to the dependence of parasites and their potential vectors or intermediate hosts on numerous environmental factors (e.g., availability and type of hosts (e.g., humans, domesticated or wild animals), habitat conditions such as temperature, humidity, availability and type of waters (e.g., running or stagnant ones), etc.). For instance, malaria, the parasitic disease par excellence of the African continent, provides a (hopefully) compelling example of a condition that needs to be tackled through One Health efforts, due to the zoophilic behaviour of several competent mosquito vectors [57,58]. This is the approach taken by recent investigations aiming at treating cattle and other livestock with ivermectin or macrocyclic lactone-based “endectocides”^4^ in order to reduce the fitness of competent malaria vectors feeding on them [59]. Similarly, the application of pyrethroid-based insecticides (e.g., deltamethrin, permethrin and lambdacyhalothrin) or arthropocides to cattle not only aims to control livestock-specific ectoparasites but also to reduce populations of zoophilic mosquitoes responsible for malaria transmission [57,60]. These methods could be considered as complementary tools that could help reduce malaria incidence in a given area, although alone they may not suffice to eradicate the disease. It is nonetheless essential that control campaigns consider all hosts on which competent mosquitoes feed and the outdoor component of malaria transmission.

Other major parasitoses of the African continent are ascribed in the group of the so-called Neglected Tropical Diseases (NTDs), of which Africa bears ~40% of the world’s burden [61]. These are typical diseases of poverty, highly endemic in rural areas, not coincidentally where hygiene conditions and water sanitation are poor and where tight cohabitation between humans and animals occur [61]. Out of the 20 NTDs recognised by the WHO, 19 occur in Africa, with 11 (nearly 60%) of them being of parasitological aetiology. Of these, at least seven include zoonotic agents (see also Table 1). Although not involving a parasitic causative agent, NTDs like dengue and chikungunya still require parasitic arthropods (i.e., mosquitoes of the genus *Aedes* spp.) as vectors [62,63], further highlighting the relevance of parasitological and entomological expertise in the study, management and control of most NTDs in Africa and elsewhere (Table 1).

Undoubtedly noteworthy in the area of One Health are also the so-called Neglected Zoonotic Diseases (NZDs) [64], that are deemed as “neglected” due to under-reporting on their occurrence, leading to an underestimation of their relevance to policy-makers and donors [65]. The NZDs comprise a subgroup of NTDs (i.e., echinococcosis, food-borne trematodiases, human African trypanosomiasis, leishmaniasis, rabies, schistosomiasis and taeniasis/cysticercosis,), all of which except rabies are of parasitic aetiology. Currently, rabies, echinococcosis, food-borne trematodiases and taeniasis/cysticercosis are considered as priority NZDs by the WHO Department of Control of NTDs [64]; the fact that three of them are parasitic infections further highlights the topicality and heavy burden of parasites in the realm of zoonoses. In addition, zoonotic causes of non-malarial febrile illnesses such as anthrax, brucellosis and leptospirosis are also recognised by the WHO to be of growing importance and are therefore monitored accordingly [64].

The control of NTDs is a firm component of the UN 2030 Agenda for Sustainable Development, as reflected in its Sustainable Development Goal (SDG) target 3.3 aiming, by 2030, to “end the epidemics of AIDS, tuberculosis, malaria and NTDs (…)” [66]. In the past decade, some substantial progress was made in these regards, with 31 countries having succeeded in eliminating at least one NTD, eight of them being in Africa [67]. However, despite these results, many of the targets set by the WHO in the 2020 roadmap were not met, in Africa and other endemic regions [61]. In particular, the overall control of NTDs was negatively affected by the disruptions caused by the COVID-19 pandemic in the year 2020, with major hindrances being recorded in the delivery of health services [68]. Accordingly, a new NTDs roadmap was defined for the period 2021–2030, identifying critical gaps and actions required in order to prevent, control, eliminate or eradicate the 20 target diseases and disease groups [61]. Importantly, the new agenda fully acknowledges the value of adopting an integrated One Health strategy for NTDs, promoting stronger multisectoral collaborations among agriculture, livestock, wildlife, environment, food safety, health and other Ministries [61]. Such a strategic development derives from the consideration that all zoonotic NTDs, in order to be fully controlled in human populations, need to be managed appropriately and possibly synergistically in animal hosts and/or reservoirs. Notably, tackling NTDs involving livestock species in their epidemiology (e.g., parasitoses like echinococcosis, human African trypanosomiasis, schistosomiasis and taeniasis/cysticercosis), can provide dual benefits in terms of human and economic development, by reducing risks of zoonotic infections, on one hand, and improving communities’ livelihood, through better animal health and productivity, on the other [65,69].

Moreover, the African continent is also affected by a number of vector-borne zoonotic infections caused either by viruses (e.g., Crimean-Congo Haemorragic Fever (CCHF), Rift Valley Fever (RVF) and Yellow Fever) [70,71,72] or by bacteria (e.g., relapsing fever borrelioses and several rickettsioses (e.g., by *Rickettsia aeschlimannii*, *Rickettsia africae*, *Rickettsia conorii*, *Rickettsia felis*, *Rickettsia massiliae* and *Candidatus* Rickettsia asemboensis, etc.)) [73,74,75,76], which require parasitological and entomological expertise to be effectively addressed, due to their vectoral components (e.g., ticks in the case of CCHF, relapsing fever borrelioses and rickettsioses; mosquitoes in the case of RVF; fleas in the case of *R. felis* and *C.* Rickettsia asemboensis). Indeed, recognising the parasitological/entomological constituents in the epidemiology of these infections is essential as it helps identifying potential arthropod or vertebrate reservoirs, thus ultimately preventing and controlling the diseases in humans and animals. In Uganda, for example, when it was first encountered, in 2013, CCHF was initially feared to be the more deadly Ebola [77], which has occasionally crossed the country’s border with the Democratic Republic of the Congo [78,79]. Ever since, CCHF has occurred in the country in the form of outbreaks within or in the proximity of the “cattle corridor”, a region spanning northeastern, central and southwestern districts, known indeed for the density of its cattle herds [80,81]. With most cases being associated with exposure to bites of ticks (especially *Hyalomma* spp.) who have fed on viraemic cattle or ruminant hosts [80], the effective control of this infection could be achieved through the roll-out of strategic targeted tick control programmes and the sensitisation of the general public and farming communities on the epidemiology of CCHF. This would require a concerted initiative to be conceived and implemented by several Ministries in coordination (e.g., in the case of Uganda, a partnership between the Ministry of Health and the Ministry of Agriculture, Animal Industry and Fisheries) [80]. Considering that the same cattle corridor witnesses sporadic cases of mosquito-borne RVF [81], whose reservoir is also represented by ruminants [71], One Health efforts in this area should opt for the use of “arthropocidal” molecules, halting both ticks and mosquitoes, rather than merely “acaricidal” products (controlling ticks) or “insecticidal” ones (controlling insects such as mosquitoes).

Moreover, the role of “companion” animals or non-livestock species, such as dogs and cats, is also critical for certain parasitic zoonoses in Africa. In addition to their involvement in a number of NTDs (e.g., dogs as final hosts of *Echinococcus granulosus* and *Dracunculus medinensis*, and reservoirs of *Leishmania* spp.), these species are also implicated in the epidemiology of several zoonotic geohelminths such as *Toxocara* spp., *Ancylostoma* spp. (e.g., *Ancylostoma caninum* and *Ancylostoma braziliense*) and *Strongyloides stercoralis*, and cestodes (e.g., *Dipylidium caninum*), entailing direct or indirect transmission through an intermediate host (e.g., fleas and *Trichodectes* lice in the case of *D. caninum*) [76,82]. The frequent free-roaming behaviour of dogs and cats, typically in rural milieus across Africa [83], coupled with the usually poor hygiene and water sanitation of such settings, can particularly favour the spread of these parasitoses and enhance exposure risks in humans and other animal species. In addition, with the size of middle class rising in the continent [84], ownership of dogs and cats as “pets” may potentially increase in urban areas in the next decades, highlighting the importance of parasite control also in urban and peri-urban sites, where encounters between “owned” and “free-roaming” animals, and their excretions, may occur.

Furthermore, the contribution of wildlife needs also to be considered, either as definitive (e.g., lions and hyenas for *Echinoccocus felidis*) or intermediate hosts (e.g., warthogs for *E. felidis*) or even reservoirs (rodents in the case of *Schistosoma* spp.) of zoonotic parasites [85,86]. Therefore, in order to be fully and durably effective, One Health initiatives tackling parasitic conditions should take into account all final, intermediate, potentially vector and even paratenic or transport hosts involved in life cycles of target aetiological agents. Overlooking even a single host species, wildlife included, can indeed cause the reappearance or impede the elimination tout court of cases of infection or disease in certain other hosts, despite generous control efforts being addressed towards them.

## 3. One Health beyond Parasitic Zoonoses

Should the One Health approach be envisaged only in the case of zoonotic parasitic or arthropod-borne infections that are shared between humans and animals? This concept paper advocates for a more comprehensive assessment of parasites and vectors in this paradigm. The health of livestock (e.g., cattle, sheep, goats, camels, poultry, pigs, donkeys, etc.) in Africa is indeed undermined by several parasites or arthropods that, although not zoonotic per se, cause major chronic deterioration and productivity losses, being responsible for low body condition scoring, poor protein conversion and, overall, scarce production (i.e., in milk, meat, eggs and skins). In the case of cattle, for instance, gastrointestinal nematodes, ticks and tick-borne infections and animal African trypanosomiasis are the most important examples to be incriminated in these regards [87]. The productivity losses that they entail are therefore inextricably connected with the continent’s food insecurity, especially considering that the largest bulk of Africa’s food production is used for local consumption [14]. With food insecurity being recognised as a global health challenge [10,55], all its major causes in the animal sources should be thoroughly investigated and tackled under the One Health lens. This applies also to animals’ and livestock parasitic infections, given the burden they pose, ultimately, to human sustenance and nutrition. As the OIE puts it, “pathogens of animal origin that are not transmissible to humans, but which have a severe impact on the production of animal protein, should not be neglected either, particularly in developing countries. In fact, they can lead to production losses and a reduction in the available food supply, leading to serious public health problems caused by food shortages and protein deficiencies” [55].

## 4. One Health Approach for Research and Development of Parasiticides

The field of research and development (R&D) of parasiticides provides perhaps the most blatant concrete examples of the One Health approach in parasitology, spanning across human and veterinary medicine. The possibly most renowned case in point in this respect is represented by the “wonder drug” ivermectin. Discovered in the late 1970s and initially developed for veterinary use as a broad-spectrum endectocide targeting gastro-intestinal nematodes and several ectoparasitic arthropods, ivermectin has then been largely employed in human medicine for the control of onchocerciasis, lymphatic filariasis (LF), certain soil-transmitted helminthiases (e.g., by *Ascaris lumbricoides* and *S. stercoralis*) and scabies [88]. This drug still represents the mainstay of two global campaigns aiming to eliminate onchocerciasis and LF, by the means of Mass Drug Administration (MDA), made possible through donations of the active ingredient (produced under the brand name Mectizan^®^) by Merck & Co., Inc. (Kenilworth, NJ, USA) (for LF, ivermectin is administered in combination with the anthelmintic albendazole, donated by GlaxoSmithKline, GSK) [89]. Recently, the use of ivermectin was proposed for MDAs in both humans and livestock as a complementary strategy to control malaria mosquito vectors [90].

Another successful example of an anthelmintic employed in both human and veterinary medicine is that of praziquantel. Also discovered in the 1970s, it is used to control cestodes and trematodes in dogs, cats, horses [91] and sometimes even cattle [92], and it represents the only currently available option for the control of schistosomiasis in humans [93]. Over the years, praziquantel has indeed been used for MDA-based preventative chemotherapy (PC) campaigns targeting school aged children in schistosomiasis-endemic areas in Africa [93].

However, risks of emergence and/or spreading of resistance or reduced efficacy in human parasites addressed towards ivermectin (i.e., in *Onchocerca volvulus*) [94,95] and praziquantel (i.e., in *Schistosoma* spp.) [96], together with the need for effective drugs and/or vaccines against NTDs and malaria, have paved the way for initiatives aiming to fill the void in R&D pipelines against these diseases. Indeed, from the early 2000s onwards, several “Product Development Partnerships” (PDPs) started being established, under the model of “Public–Private Partnerships” (PPPs). These include, among others, the Drugs for Neglected Diseases initiative (DND*i*) [97], Medicines for Malaria Venture (MMV) [98] and the Global Health Innovation Technology (GHIT) [99], focused on drug and/or vaccine development; the Foundation for Innovative Diagnostics (FIND) [100] devoted to diagnostics; the Program for Appropriate Technology in Health (PATH) [101] dedicated to vaccine R&D, primary health care and advocacy; and the Innovative Vector Control Consortium (IVCC), developing vector control tools [102]. Such PDPs are non-profit entities that collaborate with human pharmaceutical and biotech or agrochemical firms to support them in the development of solutions to address NTDs and malaria (e.g., from discovery to clinical trials and registration). Within this framework, the substantial funding provided by multilateral, bilateral and philanthropic donors (e.g., Bill & Melinda Gates Foundation), allows for the “de-risking” of R&D projects that would otherwise be prohibitive for industry alone, due to the limited return on investment (ROI) that they may generate (being NTDs and malaria, by definition, the diseases of the poor) [103,104]. The execution of the London Declaration on NTDs in 2012, which gathered initially the world’s 13 leading pharmaceutical companies, generated a major momentum in the fight against NTDs, catalysing an investment of more than US$785 million to support R&D programmes [105].

For example, DND*i*’s target conditions include several parasitic NTDs that are endemic to the African continent such as leishmaniasis, sleeping sickness (trypanosomiasis), LF and onchocerciasis [106]. With respect to the latter condition, DND*i*’s efforts are currently focused on evaluating emodepside, an anthelminthic veterinary drug used to control nematodes in cats and dogs, for the development of an oral macrofilaricidal treatment for onchocerciasis in humans, to be delivered as a tablet [107]. Following successful Phase I studies in healthy volunteers, this project will soon undergo a Phase II proof-of-concept clinical trial in Ghana, aiming to assess the safety and efficacy of emodepside for people living with onchocerciasis [108].

In the veterinary field, the Global Alliance for Livestock Veterinary Medicines (GALVmed) was established in 2004 as a PDP supporting veterinary pharmaceutical companies developing drugs, vaccines and diagnostic tools for livestock diseases of poor smallholder farmers in sub-Saharan Africa and South Asia (e.g., India, Bangladesh and Nepal) [109]. Some of GALVmed’s target diseases include NZDs such as porcine cysticercosis and animal African trypanosomiasis and the zoonotic arthropod-borne RVF [110]. In the case of porcine cysticercosis, GALVmed bolstered the commercial development of a dual approach based on the first licensed cysticercosis vaccine for pigs (i.e., TSOL18), administered concurrently with a therapeutic drug (i.e., oxfendazole), used to eliminate parasitic larvae, according to a “therapeutic-prevention” strategy [111]. Following successful trials in Nepal, Tanzania, Uganda and Zambia, both products are now undergoing registration in several African countries [112]. Given the zoonotic nature of cysticercosis, this initiative is expected to bring also public health benefits to the communities administering these products to their pigs.

The examples of dewormers such as ivermectin and praziquantel show that opportunities do exist with regards to translating or even “repurposing^5^” drug discovery and development from human to veterinary health applications, and vice versa, in the area of parasitic infections. Considering the importance of nematodes and arthropod pests in agriculture, both veterinary and human pharmaceutical industries can potentially benefit from collaborations with agrochemical firms in this space. However, depending on the epidemiological settings, risks of emergence of resistance or reduced efficacy in humans due to the widespread use of a given compound or class of parasiticides in animals, not only should be anticipated, but should also be avoided, learning from the experiences with antimicrobial use. It would therefore be desirable that veterinary and human pharmaceuticals’ R&D efforts, while still synergising on potential repurposing efforts, would also deliver “complementary” therapeutic options that would allow for the sustainable and yet effective management of infections across all concerned host species, minimising the risks of resistances by relying on different modes of actions, when possible. Given the wider occurrence of parasites in animals compared to humans and considering the profitable market of veterinary parasiticides (including ectoparasiticides, endoparasiticides and endectocides) in the developed world [85], it should not be excluded that the experience generated in animal health R&D programmes could prove useful for humans too. Because of this, establishing dedicated fora of communication exchange among human and veterinary pharmaceutical firms, in the spirit of the London Declaration, may bring benefits to global public health. At the same time, potential changes of distribution patterns of certain insect-borne NTDs (e.g., dengue and chikungunya), due to global warming, may lead, in future decades, to an increase of their prevalence in more mildly temperate high-income countries [113,114]. This may provide an ROI-driven impetus prompting more R&D ventures, also in the area of vector control, for both developing and developed countries.

## 5. Perspectives from Here

Considering the review above, this concept note upholds a number of recommendations, to be taken into account when dealing with parasitic and arthropod-borne infections in Africa and beyond, in current COVID-19 times and further. Such recommendations not only are addressed to the attention of medical and veterinary parasitologists and entomologists, but also to the general public, social scientists, public health, pharmaceutical and biotech industry professionals and policy-makers.

**Figure 1:**
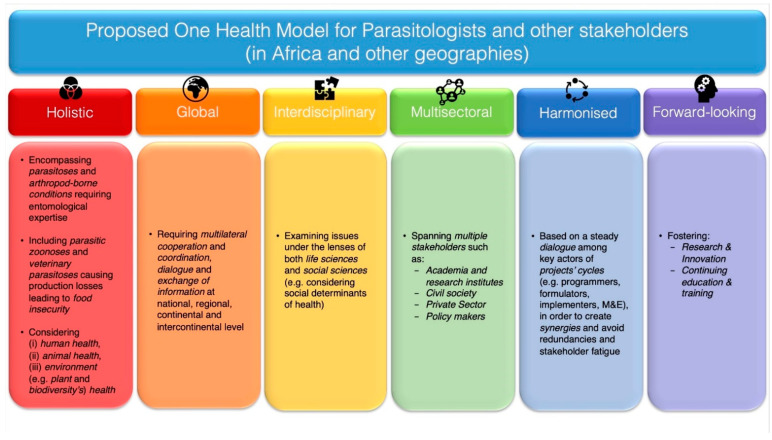
Proposed One Health model, addressed to medical and veterinary parasitologists and entomologists, as well as general public, social scientists, public health, pharmaceutical and biotech industry professionals and policy-makers, dealing with parasitic and vector-borne infections, in Africa and beyond.

This appraisal underpins that, for it to be effective, the One Health approach should be adopted or, rather, embraced, through a *holistic*, *global*, *interdisciplinary*, *multisectoral*, *harmonised* and *forward-looking* set of actions (see also Figure 1). Below will be provided elaborations for each of the adjectives used to define the One Health model here recommended.

i. The *holistic* perspective of the One Health approach here recommended encourages the involvement of parasitologists in issues pertaining not only to (i) mere parasitic conditions of humans and animals, but also to (ii) vector-borne infections caused by other pathogens (e.g., viruses and bacteria), entailing nevertheless parasitic arthropods in their epidemiology and therefore requiring entomological expertise in order to be tackled comprehensively and effectively. Moreover, the present analysis suggests that parasitologists should consider under the One Health approach not only (i) zoonoses, but also (ii) non-zoonotic veterinary parasitoses responsible for productivity losses in livestock, thus for food insecurity in their respective communities.
    With the role of agriculture being as fundamental to Africa’s ongoing and future socio-economic development, the analysis here provided underscores the indissolubility, when dealing with parasitic and vector-borne infections according to the One Health approach, of concepts such as (i) human health and (ii) animal health, as well as (iii) environment (e.g., plant and biodiversity’s) health. After all, the well-being of humans, animals and the environment is inextricably linked to agriculture and its several practices (e.g., agribusiness, agroecology, agroforestry and pastoralism) and to the management and use of wild resources, including flora, fauna and water. Accordingly, the durable insurance of the well-being of human beings and their livestock will necessarily need to encompass the preservation of habitats and biodiversity richness.
ii. The *global* nature of this effort stems from the realisation that no public health issue has a merely local dimension, as it has been clearly shown by the COVID-19 pandemic. In the case of parasitic and vector-borne infections, there is no condition that can be considered as a null threat outside where it originates, especially if naïve areas of potential novel introduction harbour suitable habitats for the parasite or its intermediate host(s) or vector(s) to develop. For example, this is evidently the case for food-borne parasitoses (e.g., echinococcosis, toxoplasmosis and trematodiases) that could be carried to significant distances through trade of food produces [115] or for infections that can reach new areas through movement of infective vectors (e.g., mosquitoes) or intermediate hosts (e.g., snails) via human-made means of transports (e.g., airplanes or ships) [116,117,118] or by dispersion of vectors through migratory birds (e.g., *Hyalomma* ticks parasitising migratory birds and posing risks of new foci of CCHF) or other parasitised hosts [118,119,120] or through travel of infected hosts [118]. In the latter case, it should however be noted that a population of competent vectors would need to occur in the area of new introduction for a vector-borne infection to successfully establish itself [118].
    Undoubtedly, practicing the One Health approach globally requires great cooperation and coordination among institutions at national, regional, continental and intercontinental level, to ensure the constant exchange of information and the continuous advancement of surveillance and response systems. This should be done with the awareness that exchanging information among countries or regions can provide mutual benefits in terms of capacity building and thus preparedness and responsiveness in all geographies involved in such a dialogue. Accordingly, improving capacity in Europe on the detection and surveillance of “exotic” vector-borne infections that are currently endemic in some parts of Africa, such as RVF, can also provide enhanced training opportunities to African scientists and researchers, who, in turn, may further potentiate their monitoring systems by exchanging views and personnel with non-endemic third countries.
iii. The *interdisciplinary* angle of this effort requires the consideration of parasitic and arthropod-borne infections under the lens of both life sciences and social sciences. Indeed, all types of control efforts can only be effective in a given area (from small to large, regardless of its size) if designed, implemented and evaluated by taking into account the social determinants of health, in which people are born, grow, live, work and age. These include factors like socio-economic status, education, neighbourhood and physical environment, employment, and social support networks, as well as access to health care of communities [121]. The involvement and “ownership” of local communities, that are the ultimate beneficiaries of interventions, whether treatments are addressed to humans or animals or the environment, should indeed be mandatory for all One Health (and beyond) interventions, dealing with all types of conditions, not only parasitic ones. Only a deep understanding of communities’ practices and customs can allow for the conception of potentially effective initiatives, which should be co-designed with recipient communities.
    For example, in the early 2010s, conversations with cattle keepers from northern Uganda (i.e., districts of Kaberamaido and Dokolo), reporting of being not rarely bitten by “colourful” ornate ticks (i.e., *Amblyomma* spp.) led to documenting for the first time in the country the occurrence of the zoonotic pathogen *R. africae*, causative agent of African Tick-Bite Fever (ATBF) [122], a condition often misdiagnosed with malaria- or flu-like syndromes [123]. Such finding highlighted the risk of exposure to ATBF of rural communities in northern Uganda, underpinning the importance of raising awareness on this rickettsiosis, particularly among persons handling cattle (e.g., herders, veterinarians and paraveterinarians) as well as among physicians practicing in these areas, and those who care for returning travellers [122]. It is thanks to farmers’ viewpoints that this investigation could be started, and such a public health risk could be unveiled.
iv. The *multisectoral* nature of the approach here recommended entails the participation in One Health initiatives of all stakeholders potentially concerned, including civil society, academia, industry, institutions and their policy-makers. All parties’ contribution is essential for interventions to be successful. Academic parasitologists should therefore strive for engaging with civil society any time the investigations that they conduct may have possible repercussions on the latter. With data in hand, parasitologists as other scientists in the field of One Health, should engage in societal debate and render their research rationales, methodologies, findings and recommendations intelligible not only to the general public, but also to administrators. To some extent, the COVID-19 pandemic has shown that concepts such as antigenic or serological testing or even that of One Health itself, can become more widely accessible than they used to be beforehand, out of necessity. At the same time, academics and industry actors should proactively seek to collaborate with each other. The contribution of the private sector (e.g., pharmaceutical/biotech/vector control industries) is indeed essential in the fight against parasitic and vector-borne infections, as it allows to deliver “ready to use” solutions such as drugs, vaccines, insecticides and diagnostic tools. At the same time, serendipities happening in laboratories at universities and research institutes can lead to breakthrough discoveries that could be ultimately turned into “actual products”, responding to unmet needs on the ground, through win-win partnerships with the private sector.
v. With multiple programmes being often conducted concurrently in neighbouring, if not overlapping, geographic areas, addressing either the same or different diseases, there is a need for *harmonised* actions. These would be possible through the establishment of a steady dialogue among key actors of projects’ cycles, including programmers and formulators (e.g., donors, local authorities and communities), implementers (e.g., funding grantees, principal investigators, programme coordinators, etc.) and monitoring and evaluation teams. Creating, whenever possible, synergies between incoming projects and previous and/or concomitant initiatives can allow to optimise results (i.e., outputs and outcomes) and minimise possible redundancies and “stakeholder fatigue”, for the sake of the common good. With global health gaining presumably increasingly more political attention in the wake of the COVID-19 pandemic [124,125,126], prioritising interventions based on burden of diseases (e.g., through Disability-Adjusted Life Years (DALY)) is undoubtedly an important instrument for agenda setting. In this view, the availability of reliable data, generated through robust methodologies and thorough analyses, is essential.
vi. Finally, for it to be “ever topical” and effective, the One Health approach should also be *forward-looking*, and rely on institutional policies fostering (a) research and innovation, both at public and private level, and (b) continuing education and training in parasitology and entomology. Only through constant R&D efforts, entailing collaborations among academia, industry and PDPs, it can be hoped that more parasitic and arthropod-borne conditions of humans and animals or both, NTDs included, could be effectively controlled in the future. Fostering research and innovation as well as manufacturing capacity locally in Africa, not only could prove logistically practical and ultimately cost-effective, considering these efforts are addressed to endemic conditions of the continent, but can also provide the African burgeoning youth with major employment opportunities. This would also require tailored curricula to be put in place at local African universities. The know-how built by the Institut Pasteur de Dakar, only centre in Africa able to produce a yellow fever vaccine [127] and soon to produce vaccines against COVID-19 [128], as well as the institution of the University of Global Health Equity in Rwanda [129] and the One Health Research, Education and Outreach Centre in Africa (OHRECA) in Kenya [130] and the Africa One Health University Network (AFROHUN) [131] are just some encouraging examples in this respect, among other ongoing initiatives. Importantly, given the centrality of youth in education and the fundamental contribution of women scientists to Africa’s development [132], investing in research, innovation and training in parasitology and entomology can have an immensely empowering role and contribute to the overall attainment of the United Nations’ 2030 Agenda’s SDG #4 (“quality education”) and SDG #5 (“gender equality”).

## 6. Conclusions

From this analysis, it may appear evident that tackling parasitic conditions and arthropod-borne infections of humans and animals through the One Health approach can tangibly help the attainment of SDGs pertaining to poverty alleviation, food security and health, such as SDG #1 (“no poverty”), SDG #2 (“zero hunger”) and SDG #3 (“good health and well-being”). Furthermore, managing these infections as holistically and collaboratively as advocated in this paper may also allow the fulfilment of other interconnected SDGs, such as SDG #6 (“clean water and sanitation”) (e.g., with special regards to water-borne parasitic conditions such as schistosomiasis, trematodiases and also a number of arthropod-borne infections requiring running or stagnant waters for the development of arthropod vectors), SDG #8 (“decent work and economic growth”), SDG #10 (“reduced inequalities”), SDG #15 (“life on land”), SDG #17 (“partnerships for the goals”) and, indeed, SDG #4 and SDG #5 aforementioned.

All in all, the approach here proposed is consistent with the concept of “Planetary Health” [133], according to which the potential participation of the United Nations Environment Programme (UNEP) in the FAO–OIE–WHO Tripartite collaboration may further strengthen the overall governance of One Health initiatives in Africa and at a global scale [134]. Despite their heavy burden, parasitic and arthropod-borne infections are still one part of a large number of issues that need to be managed under the One Health lens in Africa, including, among others, antimicrobial resistance, rabies, Ebola and COVID-19. With parasitoses usually lasting longer than the time of an “epidemic”, addressing these effectively could allow long(er)-term investments in sustainable resilient health systems, that could, in turn, enhance preparedness and responsiveness, whenever needed, vis-à-vis other public health challenges. Let us roll up our sleeves.

## Figures and Tables

**Table 1:** NTDs known to occur in Africa, with emphasis on those that are of parasitic aetiology and/or arthropod-borne and/or of zoonotic importance. (Cells are coloured in grey every time the criteria from the respective columns are not met for each given NTD and causative agent).

Neglected Tropical Diseases (NTDs)	Causative Agent(s)	Parasitic Aetiology	Arthropod-Borne	Zoonotic
Buruli ulcer	*Mycobacterium* *ulcerans*			
Dengue and chikungunya	dengue virus (DENV) chikungunya virus (CHIKV)		✓	✓
Echinococcosis (hydatidosis)	*Echinococcus* *granulosus* complex	✓		✓
Food-borne trematodiases	*Fasciola* spp.; *Paragonimus* spp.	✓		✓
Guinea worm disease (Dracunculiasis)	*Dracunculus medinensis*	✓		✓
Human African trypanosomiasis (sleeping sickness)	*Trypanosoma* *brucei*	✓	✓	✓
Leishmaniasis	*Leishmania* *infantum*; *Leishmania* *donovani*; *Leishmania major*; *Leishmania aethiopica*; *Leishmania tropica*	✓	✓	✓
Leprosy (Hansen disease)	*Mycobacterium leprae*			
Lymphatic Filariasis	*Wuchereria bancrofti*	✓	✓	
Mycetoma	*Madurella* spp.; *Streptomyces* spp.; *Actinomadura* spp.; *Exophiala* spp.			
Onchocerciasis (River blindness)	*Onchocerca volvulus*	✓	✓	
Rabies	Rabies virus (RV)			✓
Scabies	*Sarcoptes scabiei* var. *hominis*	✓		
Schistosomiasis (Bilharziasis)	*Schistosoma haematobium*; *Schistosoma mansoni* and hybrid forms	✓		✓
Snakebite envenoming	Several toxins in snakes’ venoms			
Soil-Transmitted Helminthiases	*Ascaris lumbricoides* and *Strongyloides stercoralis* (roundworms); *Trichuris trichiura* (whipworm); *Necator americanus* and *Ancylostoma duodenale* (hookworms)	✓		✓ (*S. stercoralis*)
Taeniasis/ cysticercosis	*Taenia solium*/ *Cysticercus cellulosae*	✓		✓
Blinding trachoma	*Chlamydia* *trachomatis*			
Endemic treponematoses (yaws)	*Treponema* spp.			

## Author Statement

This paper was written as part of the proceeding of an invited keynote talk held at the ESDA/SoIPa Conference on 19 June 2021. This article was conceived with deep attachment and affection towards the African continent.

## Glossary

1. **Zoonoses or zoonotic infections**: Infections that are naturally transmitted from vertebrate animals to humans, and vice versa [64]. Zoonotic diseases are therefore conditions derived from such infections.
2. **Demographic dividend**: Economic growth deriving from shifts in a population’s age structure, mainly when the share of the working age population (15–64-year-olds) is larger than the non-working age share of the population (<14-year-olds and >65-year-olds). This can occur when declining fertility leads to a bulge in the proportion of the population entering the labour force, in the presence of suitable jobs. If this young cohort is healthy, well-educated and empowered, and has a chance for decent work, it can accelerate sustainable development in the course of a generation [135].
3. **Parasitologists**: Scientists devoted to the study of internal and/or external parasites (i.e., endoparasites (e.g., protozoa, nematodes, trematodes and cestodes) and ectoparasites (e.g., parasitic arthropods), respectively). The definition employed in this text is inclusive of that of entomologists (scientists devoted to the study of insects and other classes of arthropods).
4. **Endectocides**: Antiparasitic products able to kill and control both internal (i.e., endoparasites) and external parasites (i.e., ectoparasites). Endoparasites usually controlled by endectocides include nematodes (e.g., gastrointestinal and/or bronchopulmonary nematodes), whereas target ectoparasites may vary according to the active compounds used, possibly including mange mites, one host-ticks (i.e., *Boophilus* spp.), fly larvae, etc. A typical example of endectocide is provided by products based on ivermectin and other compounds of the class of macrocyclic lactones (e.g., doramectin, moxidectin, milbemycin oxime, etc.).
5. **Repurposing (or repositioning)**: The process of finding new uses outside the scope of the original medical indication of a drug [136].
